# Genome-Wide CRISPR Screening Identifies Genetic Modulators of Amyloid Precursor Protein Processing

**DOI:** 10.3390/ijms27093926

**Published:** 2026-04-28

**Authors:** You Li, Yingjia Yao, Zitao Xu, Yufei Xiong, Cheng Zhang, Li Yu, Huiling Gao, Teng Fei

**Affiliations:** 1 Interdisciplinary Research Center for Brain-Computer Interface, Key Laboratory of Bioresource Research and Development of Liaoning Province, College of Life and Health Sciences, Northeastern University, Shenyang 110819, China; nanjiliyou@163.com (Y.L.); yaoyingjia@mail.neu.edu.cn (Y.Y.); zitaox1025@163.com (Z.X.); xiong030616@outlook.com (Y.X.); zhangchengneu@163.com (C.Z.); yu_li2017@163.com (L.Y.); gaohuiling@mail.neu.edu.cn (H.G.); 2 Foshan Graduate School of Innovation, Northeastern University, Foshan 528311, China; 3 National Frontiers Science Center for Industrial Intelligence and Systems Optimization, Northeastern University, Shenyang 110819, China; 4 Key Laboratory of Data Analytics and Optimization for Smart Industry (Northeastern University), Ministry of Education, Shenyang 110819, China

**Keywords:** Alzheimer’s disease, amyloid precursor protein, APP processing, CRISPR screen, LDHB, PIAS2, post-translational modification, cellular metabolism

## Abstract

The proteolytic processing of the amyloid precursor protein (APP) is a core pathological event in Alzheimer’s disease (AD) pathogenesis, yet the global genetic regulatory networks modulating this process have not been fully characterized. To systematically identify novel regulators of APP cleavage, we performed a genome-wide CRISPR/Cas9 knockout screen utilizing an optimized UAS-GAL4-based cellular reporter, and identified genetic modulators governing amyloidogenic and non-amyloidogenic processing. The screen uncovered distinct functional gene clusters regulating the APP, prominently involving cellular metabolism, protein modification, and vesicular trafficking. Specifically, *LDHB*, *PIAS2*, *CCDC53*, and *TRIM61* emerged as novel functional modulators. Biochemical validation confirmed that ablating these genes significantly alters the metabolic balance between sAPPα and amyloid-β (Aβ) production. Finally, integration with human AD transcriptomic datasets demonstrated that these identified modulators undergo significant dysregulation in clinics. Together, these findings establish a reporter-based functional screening framework for APP processing and identify candidate regulatory nodes linked to metabolism, protein modification, and vesicular trafficking. These candidates provide a resource for future mechanistic investigation and validation in more disease-relevant AD models.

## 1. Introduction

Alzheimer’s disease (AD) is the leading cause of neurodegenerative dementia worldwide, accounting for 60–80% of all clinical cases [1,2,3]. Its defining pathological hallmarks are the accumulation of extracellular amyloid-β (Aβ) plaques and intracellular neurofibrillary tangles composed of hyperphosphorylated tau protein [4,5]. The core constituent of amyloid plaques is the Aβ peptide, a 38–43 amino acid fragment generated via the sequential proteolytic cleavage of the amyloid precursor protein (APP), a type I transmembrane glycoprotein [6]. APP is processed by dedicated secretase enzymes through two mutually exclusive and functionally divergent pathways [7]. In the amyloidogenic pathway, APP is first cleaved by the β-site APP cleaving enzyme 1 (BACE1, β-secretase), followed by the γ-secretase complex [8]. This sequential processing releases the aggregation-prone, neurotoxic Aβ peptide, alongside the APP intracellular domain (AICD). Conversely, the non-amyloidogenic pathway is initiated by α-secretase, which cleaves the APP within the Aβ coding sequence [9]. This event directly precludes the generation of the intact Aβ peptide, instead producing the neuroprotective soluble APPα (sAPPα) fragment [10,11]. Although APP processing represents an important pathological axis in Alzheimer’s disease, the disease is now widely recognized as a multifactorial disorder that also involves neuroinflammation, microglial activation, and other interacting pathogenic processes [12,13,14,15,16]. In this context, systematic delineation of the genetic networks governing APP cleavage remains an important priority for identifying disease-modifying therapeutic targets.

Genome-wide pooled CRISPR-Cas9 knockout screening enables unbiased, high-throughput functional genomic mapping of the genetic architectures underlying neurodegenerative disease [17,18,19]. By coupling CRISPR-mediated perturbation with quantitative readouts, recent screens have successfully uncovered novel regulators of tau proteostasis, microglial inflammatory activation, and neuronal survival under AD-related cellular stress [20,21,22]. However, while these assays capture critical downstream pathological mechanisms, they cannot specifically enrich for genes that directly modulate APP proteolytic processing. This is because these assays rely on secondary phenotypic readouts (e.g., neuronal cell fitness) that are not exclusively coupled to APP cleavage [23]. Thus, identifying direct genetic modulators of APP processing requires a targeted screening strategy with a readout functionally coupled to the cleavage event.

To quantify APP proteolytic processing with single-cell resolution, the enzymatic cleavage must be directly coupled to a robust reporter system. An established strategy leverages the physiological nuclear translocation of the AICD following secretase-mediated cleavage [24]. By engineering an APP-GAL4 fusion, the liberated AICD-GAL4 fragment after cleavage translocates to the nucleus to drive the reporter gene transcription via upstream activating sequence (UAS) regulatory elements [25]. Here we engineered an optimized dual-fluorescence UAS-GAL4 reporter system and applied it in a genome-wide CRISPR knockout screen to systematically identify candidate regulators of APP processing. By integrating this reporter-based screening platform with orthogonal biochemical validation and clinical transcriptomic analysis, we establish a functional screening framework for APP processing and prioritize biologically meaningful candidates for further mechanistic investigation in disease-relevant contexts.

## 2. Results

### 2.1. A Dual-Fluorescence Reporter Enables High-Fidelity Tracking of APP Cleavage

**Figure 1:**
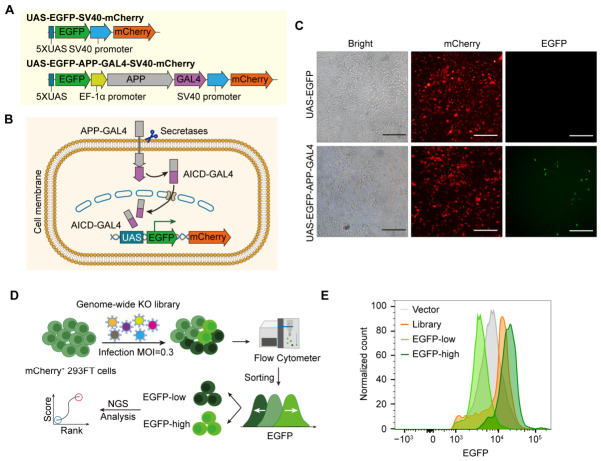
Design of the dual-fluorescence reporter system and genome-wide CRISPR screening workflow. (**A**) Schematic representation of the lentiviral dual-fluorescence reporter constructs. In the experimental vector (bottom), a constitutive EF-1α promoter drives the expression of the APP-GAL4 fusion protein, while a separate SV40 promoter drives the constitutive expression of the mCherry internal reference, with a 5×UAS element governing enhanced green fluorescent protein (EGFP) expression. The control vector (top) lacks the APP-GAL4 expression cassette. (**B**) Mechanism of reporter transactivation. Endogenous secretase-mediated cleavage releases the AICD-GAL4 domain, which translocates to the nucleus to drive EGFP transcription. (**C**) Representative fluorescence microscopy images of the monoclonal HEK293FT reporter cell line, demonstrating stable mCherry expression and dynamic basal EGFP signal. Scale bars = 500 μm. (**D**) Schematic outline of the genome-wide CRISPR-Cas9 screening pipeline, including low-MOI lentiviral library transduction, phenotypic cell sorting, and next-generation sequencing (NGS). (**E**) Representative flow cytometry histogram illustrating the EGFP fluorescence distribution of the transduced mutant cell pool. The defined sorting windows are shown for the isolation of the EGFP-low (enriched for sgRNAs targeting positive regulators of cleavage) and EGFP-high (enriched for sgRNAs targeting negative regulators of cleavage) populations.

To systematically delineate the genetic network regulating APP proteolytic processing, we generated a dual-fluorescence reporter system integrated into a single lentiviral vector (Figure 1A). For the optimized reporter, a constitutive EF-1α promoter drives the expression of a human APP-GAL4 fusion protein, while a separate SV40 promoter drives the constitutive expression of an mCherry internal reference. Upon physiological cleavage of the fusion protein by endogenous secretases at the plasma membrane, the liberated AICD-GAL4 fragment translocates to the nucleus, where it selectively binds to 5×UAS elements to drive the transcription of enhanced green fluorescent protein (EGFP) (Figure 1B). In this design, mCherry fluorescence serves as a reliable marker for successful lentiviral integration and reporter construct expression, while EGFP intensity provides a dynamic functional readout coupled to APP secretase-dependent processing.

To establish a stable screening platform, we first confirmed the intact overexpression of the engineered APP-GAL4 fusion protein in the transduced reporter cells via immunoblot analysis (Figure S1A). Subsequently, to minimize clonal heterogeneity, we isolated a monoclonal HEK293FT reporter cell line via fluorescence-activated cell sorting (FACS) for cells expressing high-level mCherry (Figure S1B). Fluorescence microscopy further validated the specificity and responsiveness of the reporter system. Control cells integrated with UAS-EGFP but not with APP-GAL4, which showed a negligible EGFP signal, confirming the low-background activity of the UAS promoter. In contrast, the established monoclonal reporter line exhibited robust mCherry expression, alongside a detectable yet heterogeneous EGFP fluorescence at basal state (Figure 1C). Crucially, this non-saturated and dynamic basal activity confirms that the engineered reporter is efficiently recognized and processed by endogenous secretases. It establishes a bidirectional dynamic range, enabling the sensitive detection of both increases and decreases in APP cleavage efficiency.

For the high-throughput functional genomic screen, the monoclonal reporter cell line was transduced with a human genome-wide CRISPR-Cas9 sgRNA lentiviral library. Transduction was performed at a low multiplicity of infection (MOI = 0.3) to ensure that the vast majority of transduced cells harbored a single sgRNA integration (Figure 1D). Following 7 days of culture to allow for sufficient cell expansion and loss of target protein expression via CRISPR-Cas9-mediated gene knockout, cells were subjected to FACS. We sorted the cellular fractions with the lowest 10% (EGFP-low) and highest 10% (EGFP-high) EGFP fluorescence intensities (Figure 1E). The EGFP-low fraction was enriched for the loss of function of positive regulators of APP cleavage, while the EGFP-high fraction was enriched for the loss of function of negative regulators. Genomic DNA was extracted from these functionally distinct sorted populations, and next-generation sequencing (NGS) was performed to quantify sgRNA abundance and systematically identify the genetic modulators of APP proteolytic processing.

### 2.2. Genome-Wide CRISPR Screen Faithfully Recapitulates Canonical Regulators of APP Cleavage

**Figure 2:**
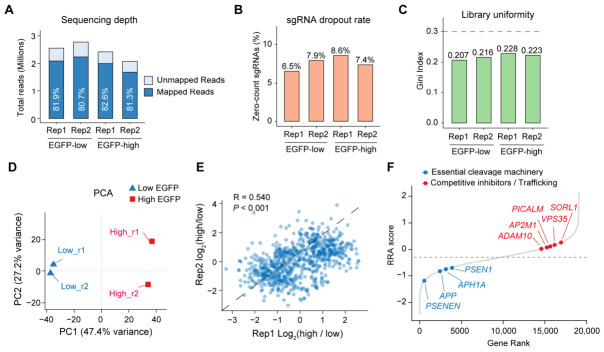
Quality control of the genome-wide CRISPR screen for APP cleavage regulators. (**A**) NGS depth, showing the proportion of mapped vs. unmapped reads across biological replicates and sorted populations. (**B**) sgRNA dropout rate quantified as the percentage of zero-count sgRNAs, confirming the maintenance of high library coverage. (**C**) Library uniformity assessed by the Gini index across the sorted samples. The dashed red line indicates the threshold of Gini index = 0.3. (**D**) Principal component analysis (PCA) showing high concordance between independent biological replicates. (**E**) Scatter plots showing the good correlation of log_2_ fold-change in normalized sgRNA abundance (EGFP-high vs. EGFP-low) between the two biological replicates. (**F**) Genes were ranked according to the tRRA scores of the screening. Canonical regulators of APP cleavage are highlighted.

To validate the performance of our screening platform, we first assessed the quality of the NGS data. Sequenced libraries exhibited high read mapping rates to the sgRNA reference library and relatively low sgRNA dropout rates for these sorted samples (Figure 2A,B). Moreover, a consistently low Gini index was observed for all samples (Figure 2C), indicating that sgRNA representation was largely maintained throughout the screening and sorting process. Thus, there was no apparent pre-existing bottleneck effect for the downstream analysis. Furthermore, the two biological replicates showed high concordance in principal component analysis (PCA) and scatter plots of sgRNA abundance between the EGFP-high and EGFP-low populations (Figure 2D,E). These data suggested a high quality of our FACS-based screening samples.

Next, we systematically identified genetic modulators driving the observed phenotypic shifts using the MAGeCK Robust Rank Aggregation (RRA) algorithm [26] (Table S1). The sgRNA abundance was compared between the EGFP-high and EGFP-low samples and a transformed RRA score was assigned to each interrogated gene. A positive score indicated sgRNA enrichment in the EGFP-high fraction (corresponding to negative regulators of APP cleavage), while a negative tRRA score indicated enrichment in the EGFP-low fraction (corresponding to positive regulators of APP cleavage). As expected, this genome-wide screen faithfully recapitulated canonical regulators of APP processing (Figure 2F), providing robust internal validation of our screening platform. For example, core components of the non-amyloidogenic α-secretase complex (*ADAM10*), along with known endosomal-lysosomal trafficking regulators of APP processing (*VPS35* [27], *SORL1* [28], and *AP2M1*), were apparently enriched in the EGFP-high population. Loss of function of these genes inhibits the non-amyloidogenic pathway or disrupts physiological APP endocytosis, shunting the APP toward amyloidogenic processing and increasing reporter signal. Conversely, the EGFP-low population was strongly enriched for the indispensable machinery required for reporter activation. This included the substrate itself (*APP*), alongside the entire core structure of the γ-secretase intramembrane aspartyl protease complex (*PSEN1*, *PSENEN*, *APH1A*, and *NCSTN*) [29]. Ablation of either the APP-GAL4 substrate or its essential cleaving proteases intrinsically abolishes GAL4 release, thereby silencing the EGFP reporter. Altogether, these results demonstrate the robustness of our screening platform which is valid to identify key functional modulators of APP cleavage.

### 2.3. Phenotypic Screening Identifies Distinct Functional Networks and Novel Regulators of APP Proteolytic Processing

**Figure 3:**
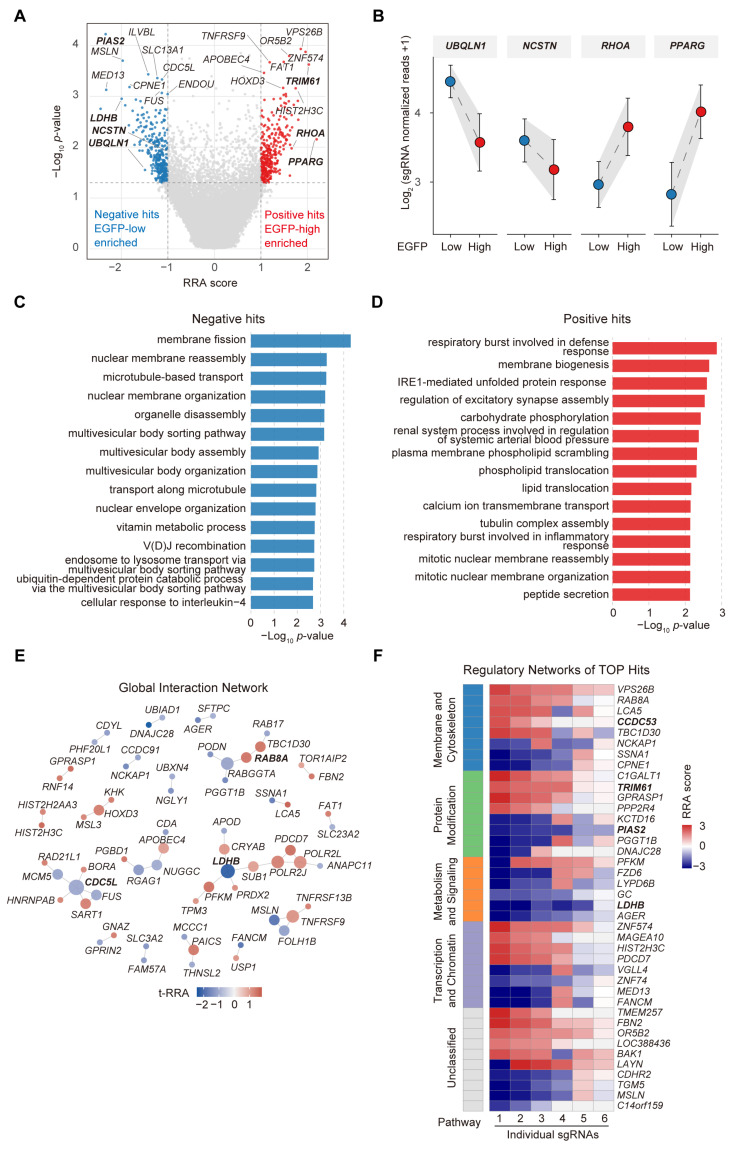
Functional genomic characterization of novel regulators of APP proteolytic processing identified via genome-wide CRISPR screening. (**A**) Volcano plot showing the defined top hits in the screen. Positive regulators of APP cleavage (negative hits enriched in EGFP-low) and negative regulators (positive hits enriched in EGFP-high) are separated by statistical significance (−Log_10_ *p*-value) and tRRA score. (**B**) Trajectory analysis of normalized sgRNA read counts showing the abundance shifts in representative canonical and novel hits (e.g., *UBQLN1*, *NCSTN*, *RHOA*, *PPARG*) between the EGFP-low and EGFP-high sorted fractions. (**C**,**D**) Gene Ontology (GO) enrichment analysis of the defined top positive and negative regulators of APP cleavage. (**E**) Protein–protein interaction (PPI) network of the top 75 high-confidence hits (enriched and depleted) constructed using the STRING database. The network topology identifies three highly interconnected functional modules involved in vesicular trafficking, transcriptional regulation, and metabolic signaling. (**F**) Gene-concept heatmap showing the regulatory directionality and phenotypic consistency across multiple independent sgRNAs for the highlighted core candidate regulators of APP cleavage.

To prioritize a set of candidate hits for downstream interpretation and validation, we applied a practical threshold (absolute tRRA score ≥ 1.0 and *p* < 0.05) (Table S1). Both positive regulators (enriched in the EGFP-low fraction) and negative regulators (enriched in the EGFP-high fraction) were identified for APP cleavage (Figure 3A). Consistent with their established biological functions [30], core components of the γ-secretase complex (e.g., *NCSTN*) and protein quality control regulators (e.g., *UBQLN1*) showed significant enrichment in the EGFP-low population at individual sgRNA levels (Figure 3B). Conversely, novel regulatory nodes involved in actin cytoskeletal dynamics (*RHOA*) and lipid/metabolic homeostasis (*PPARG*) were consistently enriched in the EGFP-high population (Figure 3B), demonstrating the ability of our platform to capture diverse upstream regulatory pathways in APP processing.

We then performed Gene Ontology (GO) enrichment analysis to define the global functional profiles of these identified APP modulators (Figure 3C,D). Positive regulators of APP cleavage were primarily associated with biological processes including “membrane fission” and “microtubule-based transport”. This functional signature is consistent with the known importance of endosomal-lysosomal sorting and axonal transport in amyloidogenic APP processing, wherein APP and BACE1 are co-transported and internalized into acidic endosomes to enable efficient Aβ generation [31]. In contrast, negative regulators of APP cleavage were enriched in functionally distinct clusters, most notably “membrane biogenesis”, “carbohydrate phosphorylation”, and the “IRE1-mediated unfolded protein response (UPR)”. Significant enrichment of the IRE1–UPR axis is consistent with a potential association between ER stress-related pathways and APP processing dysregulation [32]. Furthermore, the enrichment of carbohydrate and lipid metabolism pathways suggests that cellular energetic state and membrane-associated metabolic features may be linked to APP processing regulation.

To assess the physical connectivity and functional crosstalk between identified candidate regulators, we constructed a global protein–protein interaction (PPI) network using the top high-confidence hits (Figure 3E). Topological analysis revealed that these genetic modulators do not act as isolated effectors, but cluster into three functionally distinct and interconnected modules. The first module is associated with post-Golgi vesicular trafficking and membrane dynamics, anchored by RAB GTPase family regulators including *RAB8A* [33] and *NCKAP1* [33]. A second distinct sub-network comprises factors involved in RNA processing and chromatin organization, centered on spliceosome and transcriptional regulators such as *CDC5L* [34] and *FUS* [35]. Notably, we identified a highly interconnected metabolic and signaling cluster, driven by the core regulatory hub lactate dehydrogenase B (*LDHB* [36]) and the glycolytic enzyme phosphofructokinase muscle type (*PFKM* [37]). This network topology highlights a candidate metabolic module associated with APP processing, suggesting that aerobic glycolysis and lactate-related pathways may be functionally linked to APP regulation.

To prioritize targets for downstream validation, we systematically assessed the phenotypic consistency of top-ranked candidate regulators. We constructed a customized regulatory network of the most significantly enriched and depleted hits, alongside a gene-concept heatmap tracking the regulatory directionality of independent sgRNAs targeting each gene (Figure 3F).

The phenotypic robustness of these candidates was validated by consistent regulatory directionality across independent sgRNAs targeting the same gene locus. Within this regulatory framework, candidate targets are segregated into four distinct functional categories. In the membrane and cytoskeleton category, regulators of vesicular trafficking (including *RAB8A* [33] and the core node *CCDC53*), showed primarily positive enrichment scores and were therefore classified within the negative regulator group for APP cleavage. The protein modification category included ligases such as the E3 ubiquitin ligase *TRIM61* and the SUMO ligase *PIAS2* [38], which displayed divergent regulatory effects. These findings suggest that post-translational modifications (PTMs) may contribute to APP processing and warrant further mechanistic investigation.

Consistent with the global PPI network topology, the metabolism and signaling module reaffirmed *LDHB* as a core regulatory hub. *LDHB* showed consistent negative enrichment scores across all targeting sgRNAs, supporting its classification as a positive regulator of APP cleavage, alongside *PFKM*. Finally, the transcription and chromatin category included structural and epigenetic regulators (e.g., *HIST2H3C* and *MED13* [39]), complementing the multi-dimensional regulatory landscape of APP proteolytic processing.

### 2.4. Biochemical and Clinical Validation of Core Genetic Regulators of APP Processing

**Figure 4:**
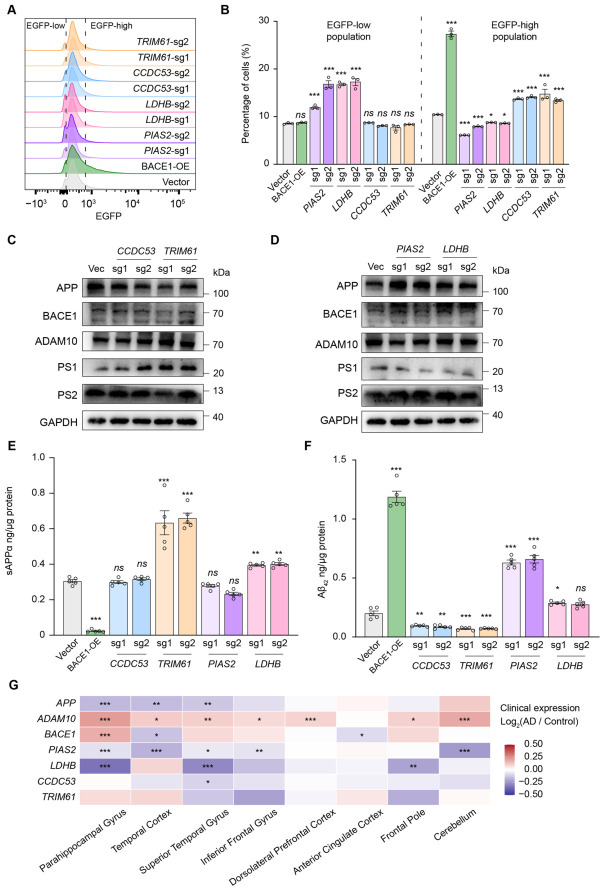
Biochemical, cellular, and clinical validation of core APP processing regulators. (**A**,**B**) Flow cytometry-based quantification showing changes in the EGFP/mCherry reporter profile following CRISPR-Cas9-mediated knockout, using two independent sgRNAs per gene for *PIAS2*, *LDHB*, *CCDC53*, and *TRIM61*. BACE1 overexpression (OE) serves as a positive control for amyloidogenic APP processing, and a non-targeting sgRNA serves as the negative control (**A**). Quantification of the percentage of cells in the EGFP-low and EGFP-high populations across independent biological replicates (**B**). (**C**,**D**) Representative immunoblots assessing the steady-state protein levels of full-length APP, key secretases (BACE1, ADAM10, and γ-secretase components Presenilin 1 (PS1) and Presenilin 2 (PS2)), and the GAPDH loading control in HEK293FT cells, following CRISPR-Cas9-mediated knockout with two independent sgRNAs per gene (**C**): CCDC53, TRIM61; (**D**): PIAS2, LDHB). (**E**,**F**) ELISA quantification of the secreted neuroprotective sAPPα fragment (**E**) and amyloidogenic Aβ_42_ peptide (**F**) in conditioned media from each knockout cell line. Data are presented as mean ± SD (*n* = 5). Statistical significance was calculated using a two-tailed Student’s *t*-test (* *p* < 0.05, ** *p* < 0.01, *** *p* < 0.001). (**G**) Cross-regional clinical transcriptomic analysis showing the expression dysregulation (Log2(AD/Control)) of validated candidate regulators across multiple brain regions in human AD cohorts. Data are derived from the AMP-AD Agora portal. Significant transcriptional dysregulation is observed in pathologically vulnerable brain regions (e.g., parahippocampal gyrus, temporal cortex) compared to the pathologically resilient cerebellum. Statistical significance was calculated using a two-tailed Student’s *t*-test (* *p* < 0.05, ** *p* < 0.01, *** *p* < 0.001, *ns*, not significant).

To experimentally validate the screening hits, we knocked out four candidate genes (*PIAS2*, *LDHB*, *CCDC53*, and *TRIM61*) using two independent sgRNAs per gene (Figure S2A–D). We then performed flow cytometry analysis of reporter cells transduced with gene-specific sgRNAs, alongside a BACE1 overexpression control and the γ-secretase inhibitor DAPT (GSI-IX) or the BACE1 inhibitor verubecestat controls to induce or inhibit APP processing, respectively (Figure S2E–G). Quantitative analysis of fluorescence signals successfully recapitulated the bidirectional changes in EGFP reporter output observed in the genome-wide screen, as well as for known controls (Figure 4A,B and Figure S2F,G). CRISPR-Cas9-mediated knockout of *PIAS2* or *LDHB* significantly increased the percentage of cells in the EGFP-low population, whereas knockout of *CCDC53* or *TRIM61* significantly increased the EGFP-high population.

To confirm that these candidate regulators directly modulate endogenous APP processing, we assessed steady-state protein levels of the APP and its associated secretase machinery. Western blot analysis revealed a consistent decrease in full-length APP in *TRIM61* or *CCDC53* knockout cells (Figure 4C). Conversely, knockout of *PIAS2* or *LDHB* increased full-length APP levels (Figure 4D). Interestingly, some core components of secretases (e.g., presenilin 1, PS1) were also changed upon specific gene ablation (Figure 4C,D). To further quantify the proteolytic outcomes, we performed ELISA to measure the absolute secretion levels of the soluble APP ectodomain (sAPPα) and Aβ_42_ in conditioned media from each group (Figure 4E,F). As sAPPα and Aβ are the mutually exclusive end products of the non-amyloidogenic and amyloidogenic processing pathways, respectively, these biochemical assays provide critical functional evidence for the physiological relevance of these candidate regulators. Strikingly, knockout of *CCDC53* or *TRIM61* significantly reduced Aβ_42_ secretion, with *TRIM61* knockout concurrently driving a significant increase in secretion of the neuroprotective sAPPα fragment. Conversely, targeted knockout of the SUMO ligase *PIAS2* and the metabolic hub gene *LDHB* significantly increased Aβ_42_ secretion, consistent with their roles as positive regulators of APP processing. Interestingly, *LDHB* knockout also led to increased sAPPα secretion, suggesting that *LDHB* might accelerate general APP processing. Therefore, these data support the conclusion that knockout of these candidate genes alters APP-related outputs and the balance between amyloidogenic and non-amyloidogenic processing. However, these measurements do not by themselves distinguish whether the underlying effects arise primarily from changes in APP abundance, protein stability, intracellular trafficking, or proteolytic processing.

Finally, to assess the clinical and translational relevance of these in vitro findings, we analyzed regional brain transcriptional profiles of our validated APP processing regulators in human AD patient cohorts. Using the Accelerating Medicines Partnership-Alzheimer’s Disease (AMP-AD) Agora transcriptomic portal [40], we analyzed the expression of the four hits alongside canonical AD pathway drivers (*APP*, *BACE1*, *ADAM10*) (Figure 4G). Compared between AD and normal states, these interrogated genes showed complex but significantly aberrant expression changes in pathologically vulnerable brain regions, confirming their clinical relevance. For example, the neuroprotective α-secretase *ADAM10* showed widespread significant upregulation, likely reflecting a compensatory cellular response to increasing amyloid burden and associated gliosis in the AD brain. Building on this established clinical context, cross-regional expression analysis identified significant directional concordance for our top validated regulators. Most notably, *PIAS2* and *LDHB*, the two genes whose knockout significantly increased Aβ_42_ secretion, were highly downregulated (*p* < 0.001) in brain regions vulnerable to early AD tauopathy and cognitive decline, including the parahippocampal gyrus and temporal cortex. This directional concordance provides supportive transcriptomic evidence for the potential disease relevance of *PIAS2*- and *LDHB*-associated pathways in AD, although it does not establish causality.

## 3. Discussion

The present study reports the development and validation of a dual-fluorescence CRISPR screening platform for systematic identification of candidate regulators of APP processing. By coupling a UAS-GAL4-based AICD translocation reporter with a constitutive mCherry internal reference, this system enables bidirectional phenotypic enrichment in a cleavage-coupled screening format. Rather than providing a complete mechanistic dissection of each identified hit, the present work is intended to generate a prioritized functional map for subsequent validation in neuronal and in vivo AD-relevant systems. Compared to prior CRISPR-based studies on APP processing by isolating edited monoclonal cell lines and directly measuring Aβ production [41], our work leverages the ease and high-throughput of a pooled genome-wide loss of function screening platform coupled to a dual-fluorescence, cleavage-coupled APP-GAL4 reporter, thereby enabling bidirectional enrichment of candidate regulators of APP processing. Using this screening platform, integrated with orthogonal biochemical validation and clinical transcriptomic analysis, we identified candidate regulatory modules associated with cellular metabolism, protein quality control, and vesicular trafficking, thereby providing a resource for further mechanistic and disease-relevant investigation of APP processing in AD. Although the pharmacological validation experiments (using known APP processing inhibitors) further strengthen the linkage between reporter output and APP secretase-dependent processing, the reporter signal may still integrate upstream effects with APP abundance, trafficking, or other regulatory steps affecting APP homeostasis.

A key finding of this study is the identification of *LDHB* and associated metabolic enzymes as candidate regulators of APP processing. While bioenergetic deficits are a well-established hallmark of the AD brain [42], our screening and validation data suggest a potential functional association between lactate metabolism and APP processing [43]. Significant enrichment of sgRNAs targeting *LDHB* in the EGFP-low population, together with its downregulation in AD-vulnerable brain regions (e.g., the parahippocampal gyrus), points to a candidate metabolic module that may be relevant to APP regulation. Notably, a recent study demonstrated that lactate directly modifies APP via lysine lactylation (e.g., at K612), a post-translational modification that significantly ameliorates amyloid pathology and cognitive decline [44]. Building on this emerging paradigm, one possible explanation for *LDHB* action is that its malfunction disrupts intracellular lactate metabolism which subsequently affects APP and/or APP processing factors via direct protein lactylation. Alongside potential shifts in intracellular redox state or endosomal pH, this direct metabolic modification could dynamically modulate secretase activity or APP vesicular trafficking. These potential mechanisms will be interrogated in detail in future studies.

Furthermore, the identification of *PIAS2* and *TRIM61* points to a potential role for PTM-related regulation in APP processing. While SUMOylation and ubiquitination are known to modulate transmembrane protein stability [45,46], our results suggest that these PTM-related regulators may influence the balance between amyloidogenic and non-amyloidogenic APP processing. The opposing effects observed after *PIAS2* and *TRIM61* knockout on sAPPα and Aβ_42_ secretion raise the possibility that PTM-associated pathways may affect APP stability, intracellular trafficking, or processing. However, the precise molecular mechanisms and the relevant modification events remain to be determined.

Cross-regional transcriptomic analysis in human AD cohorts provides supportive transcriptomic context for the potential relevance of these regulators in human disease [47]. Specifically, the transcriptional downregulation of these genes in highly vulnerable brain regions, such as the temporal cortex, suggests that their loss of function may contribute to region-specific amyloid accumulation in the AD brain. However, these data remain correlative and do not establish causal relationships in disease progression.

In conclusion, this study establishes a functional screening framework for identifying candidate regulators of APP processing. However, several limitations of the present study should be acknowledged. First, the primary genome-wide screen and subsequent biochemical validations were performed exclusively in the immortalized HEK293FT cell line. Accordingly, the findings should be interpreted as candidate discoveries derived from a non-neuronal screening platform rather than as direct evidence of neuronal relevance. Further investigations are required to validate these findings in neuronal cell lines (e.g., SH-SY5Y) or more physiologically relevant cell models, such as human induced pluripotent stem cell (iPSC)-derived neurons. Second, the current findings are derived from in vitro cellular models. Further in vivo validation in transgenic AD mouse models is required to evaluate the systemic and behavioral effects of modulating these targets. Third, while we have established robust functional consequences for several representative novel regulators, the precise underlying molecular mechanisms, including direct biochemical interaction interfaces, specific PTM sites on APP, and the exact spatiotemporal dynamics of APP vesicular trafficking, remain to be fully elucidated. Despite these limitations, our present study provides a useful platform and candidate resource for future mechanistic and disease-relevant investigation of APP processing in AD.

## 4. Materials and Methods

### 4.1. Cell Culture and Reagents

The human embryonic kidney cell line HEK293FT (CRL-1573) was obtained from the American Type Culture Collection (ATCC). Cells were cultured in Dulbecco’s Modified Eagle Medium (DMEM; VivaCell Biosciences, Shanghai XP Biomed Ltd., Shanghai, China, #C3114-0500) supplemented with 10% fetal bovine serum (FBS; ExCell Bio, Suzhou, China, #FSP500) and a 1% penicillin-streptomycin solution (VivaCell Biosciences, #C3420-0100). All cultures were maintained at 37 °C in a humidified atmosphere with 5% CO_2_. When reaching 80–90% confluence, cells were washed with phosphate-buffered saline (PBS; BI, #02-024-1ACS) and passaged using TrypLE Express Enzyme (Gibco, Waltham, MA, USA, #12563029) to minimize proteolytic damage to cell surface proteins.

### 4.2. Plasmid Construction and Dual-Fluorescence Reporter Design

We engineered a dual-fluorescence reporter system in a single lentiviral backbone to systematically monitor APP processing. This vector (pHAGE-5×UAS-EGFP-EF1α-APP-GAL4-SV40-mCherry) contains three independent expression cassettes to minimize transcriptional crosstalk. The sensor cassette, encoding a human APP transmembrane domain fused to the GAL4 transcription factor, was driven by a constitutive EF-1α promoter. A separate SV40 promoter drives the constitutive expression of a mCherry fluorescent signal, which serves as a cleavage-independent marker associated with reporter construct expression. This configuration facilitates interpretation of EGFP-based reporter changes by providing a cleavage-independent companion signal associated with reporter construct expression. The responder cassette contains a 5×UAS to drive the expression of EGFP. Upon sequence-specific binding of the cleaved APP intracellular domain (AICD)-GAL4 fusion protein, the UAS element initiates EGFP transcription. All plasmids were constructed using Gibson Assembly seamless cloning (TIANGEN, Beijing, China, #VI201), and the final construct was verified by Sanger sequencing. The primers for molecular cloning are listed in Table S2.

### 4.3. Generation of the Monoclonal Reporter Cell Line

To minimize clonal heterogeneity and establish a high-fidelity system for genome-wide screening, we used a FACS-based clonal amplification strategy. Following lentiviral transduction and the stable integration of the dual-fluorescence reporter construct into HEK293FT cells, single-cell sorting was performed using a BD FACSAria Fusion flow cytometer. Cells with high and stable mCherry-positive (mCherry^+^) fluorescence were first isolated to ensure robust construct expression. Single cells from the mCherry^+^ population were sorted into 96-well plates at one cell per well for clonal expansion. Expanded monoclonal lines were re-analyzed by flow cytometry, and a single clone with dynamic, non-saturated basal EGFP expression and stable mCherry retention was selected for all downstream screening experiments.

### 4.4. Lentiviral Library Packaging and Transduction

The primary genome-wide screen was performed using the human H3 CRISPR knockout library (Addgene, Watertown, MA, USA, #133914), which contains 117,606 sgRNAs targeting over 18,000 human protein-coding genes in the pLentiCRISPRv2-Puro backbone. To preserve optimal sgRNA representation, the library plasmid pool was amplified in electrocompetent *E. coli* and purified using an EndoFree Plasmid Maxiprep Kit (TIANGEN, #DP117). For lentiviral production, HEK293FT cells seeded in 15 cm dishes were co-transfected with the sgRNA library plasmid (20 μg), the packaging plasmid pCMVR8.74 (15 μg), and the envelope plasmid pMD2.G (6 μg) using the Neofect DNA Transfection Reagent (Neofect (Beijing) Biotech Co., Ltd., Beijing, China, #TF20121201). The medium was replaced 16 h post-transfection, and viral supernatants were harvested at 48 h and 72 h post-transfection. The collected medium was centrifuged at 1580× *g* for 5 min to remove cellular debris, aliquoted, and stored at −80 °C. Viral titer was determined by puromycin selection in HEK293FT cells. For the genome-wide screen, the monoclonal reporter cell line was transduced with the pooled lentivirus in the presence of 6 μg/mL polybrene at a low multiplicity of infection (MOI) of 0.3 to ensure that most of the transduced cells harbored a single sgRNA integration.

### 4.5. Genome-Wide CRISPR-Cas9 Screen and FACS

Following lentiviral transduction, the mutant cell pool was treated with 2 μg/mL puromycin for 7 days to eliminate non-transduced cells. After antibiotic selection, surviving cells were recovered in standard medium for 2 days, then expanded for an additional 5 days to ensure complete CRISPR-Cas9-mediated genome editing and loss of target protein expression. For phenotypic sorting, the expanded cell pool was harvested and analyzed using a BD FACSAria Fusion cell sorter. Viable cells were directly stratified based on their EGFP fluorescence intensity. The top 10% (EGFP-high) and bottom 10% (EGFP-low) of the populations were precisely sorted. To ensure >50 × sgRNA coverage per sample, a minimum of 1 × 10^7^ cells per sorted population were harvested for genomic DNA extraction. Two biological replicates were performed.

### 4.6. Genomic DNA Extraction and Next-Generation Sequencing

Sorted cell pellets were lysed in an SDS-based extraction buffer (300 mM NaCl, 0.2% SDS, 2 mM EDTA, 10 mM Tris-HCl, pH 8.0) containing RNase A (50 μg) for 1 h at 65 °C, followed immediately by overnight Proteinase K (50 μg) digestion at 55 °C. Genomic DNA (gDNA) was subsequently purified via standard phenol-chloroform extraction and isopropanol precipitation. Following sequential ethanol washes, the purified gDNA was resuspended in nuclease-free water. Sequencing libraries were generated utilizing a two-step PCR protocol: the primary PCR enriched the integrated sgRNA cassettes from the gDNA, while the secondary PCR appended Illumina-compatible flow cell adapters and sample-specific barcodes. The final pooled amplicons were gel-purified and sequenced on an Illumina platform in paired-end 150 bp (PE150) mode.

### 4.7. Bioinformatics and Screen Data Analysis

Raw sequencing FASTQ files were de-multiplexed and aligned to the human H3 CRISPR library reference to generate sgRNA-level read count matrices. Quality control and statistical analysis were performed using the MAGeCK pipeline. To identify genetic regulators of APP cleavage, the Robust Rank Aggregation (RRA) algorithm was used to compare the EGFP-high and EGFP-low populations. The phenotypic effect of gene knockout was quantified by the transformed RRA (tRRA) score, defined as the negative base-10 logarithm of the RRA score multiplied by a directionality factor. A positive tRRA score indicated sgRNA enrichment in the EGFP-high fraction (corresponding to negative regulators of APP cleavage), while a negative tRRA score indicated enrichment in the EGFP-low fraction (corresponding to positive regulators of APP cleavage). Significant hits were prioritized using an absolute tRRA score threshold of ≥1.0 plus *p* < 0.05 (Table S1). This thresholding strategy was used as a practical prioritization criterion for downstream interpretation and validation, rather than as the sole basis for biological significance. Principal Component Analysis (PCA) and Pearson correlation coefficients were calculated to assess data quality and reproducibility between biological replicates. Gene Ontology (GO) enrichment analysis (Biological Process) was performed on significant hits using the clusterProfiler R package. Protein–protein interaction (PPI) network analysis was performed using the STRING database (v11.5) with a medium confidence threshold of 0.400, and isolated nodes with no annotated interactions were excluded. The final interactome was visualized and analyzed using the ggraph R packages.

### 4.8. Genetic and Pharmacological Validation of Reporter Responses

To validate the screening hits, individual sgRNAs targeting *PIAS2*, *LDHB*, *CCDC53*, and *TRIM61* were synthesized and cloned into the pLentiCRISPRv2-Puro backbone. The sgRNA sequences are listed in Table S2. For the positive control of amyloidogenic processing, a human BACE1 overexpression vector was constructed by cloning the BACE1 coding sequence into the pHAGE-EF1α-Blast backbone using AgeI (NEB, #R3552) and BamHI (Takara, #1605) restriction sites. Lentiviral particles for all constructs were produced and transduced into the monoclonal reporter cell line. After antibiotic selection and target gene manipulation (knockout or overexpression), shifts in the EGFP/mCherry reporter profile were quantified by flow cytometry to validate the regulatory phenotype of each target gene. In the validation experiments, biological replicates refer to independently cultured and processed cell samples prepared in separate experiments.

For pharmacological validation of the reporter system, monoclonal reporter cells were treated with the γ-secretase inhibitor DAPT (GSI-IX; Meilunbio, Dalian, China, #MB5152-1) at 10 μM or the BACE1 inhibitor verubecestat (MK-8931; Aladdin, Shanghai, China, #V413929) at 1 μM for 24 h prior to flow cytometry analysis. Following treatment, EGFP and mCherry fluorescence were analyzed using the same gating strategy as described above.

### 4.9. Immunoblotting

Cultured cells were washed twice with ice-cold PBS and lysed in RIPA buffer (Beyotime, Shanghai, China, #P0013C) supplemented with protease (Meilunbio, #MB26780) and phosphatase (Meilunbio, #MB12707) inhibitor cocktails for 15 min at 4 °C. Lysates were clarified by centrifugation at 14,000 rpm for 15 min at 4 °C, and supernatants were collected as whole-cell protein extracts. Equal amounts of protein were mixed with SDS loading buffer, resolved by 8–15% Bis-Tris polyacrylamide gel electrophoresis, and transferred onto nitrocellulose membranes (Pall Corporation, Port Washington, NY, USA, #27574625). Membranes were blocked with 5% non-fat milk in Tris-buffered saline with 0.1% Tween 20 (TBST) for 1 h at room temperature, then incubated overnight at 4 °C with the following primary antibodies: anti-LDHB (Proteintech, Wuhan, China, #14824-1-AP), anti-PIAS2 (Proteintech, #16074-1-AP), anti-TRIM61 (Proteintech, #24371-1-AP), anti-CCDC53 (Proteintech, #24445-1-AP), anti-APP (Sigma, Kawasaki, Japan, #A8717), anti-BACE1 (Bioss, Boston, MA, USA, #bs-0164R), anti-ADAM10 (Bioss, #bs-3574R), anti-PS1 (ABclonal, Wuhan, China, #A2187), anti-PS2 (CST, #8598), or anti-GAPDH (Santa Cruz, Dallas, TX, USA, #sc-25778). After three washes with TBST, membranes were incubated with the appropriate HRP-conjugated secondary antibodies (Goat anti-Rabbit IgG, Thermo Fisher Scientific, Waltham, MA, USA, #31460; Goat anti-Mouse IgG, Thermo Fisher Scientific, #31430) for 1 h at room temperature. Immunoreactive signals were visualized using enhanced chemiluminescence (ECL) substrate and imaged on a Tanon 5200 imaging system.

### 4.10. Enzyme-Linked Immunosorbent Assay (ELISA)

Absolute concentrations of secreted APP cleavage products were quantified using commercial ELISA kits. After 24 h of serum deprivation to eliminate background interference, conditioned media from HEK293FT cells with targeted genetic manipulations were collected, centrifuged to remove cellular debris, and assayed for human sAPPα and Aβ_42_ using specific ELISA kits (Cusabio, Wuhan, China, #CSB-EQ027464HU and #CSB-E10684h) according to the manufacturer’s instructions. Absorbance was read at 450 nm using a microplate reader.

### 4.11. Clinical Transcriptomic Data Analysis

To assess the clinical relevance of validated hits in human AD, multi-cohort transcriptomic data were retrieved from the Agora platform (https://agora.adknowledgeportal.org/ (accessed on 25 March 2026)), a comprehensive repository developed by the Accelerating Medicines Partnership-Alzheimer’s Disease (AMP-AD) Target Discovery Program, which hosts transcriptomic, proteomic, and metabolomic data from human AD cohorts. Region-specific bulk RNA-sequencing profiles for *PIAS2*, *LDHB*, *CCDC53*, *TRIM61*, and canonical AD pathway genes (*APP*, *BACE1*, *ADAM10*) were extracted. Patient cohorts were stratified into AD and cognitively normal control groups. Transcriptional dysregulation was quantified as the Log_2_(AD/Control) ratio across eight brain regions: parahippocampal gyrus, temporal cortex, superior temporal gyrus, inferior frontal gyrus, dorsolateral prefrontal cortex, anterior cingulate cortex, frontal pole, and cerebellum.

### 4.12. Statistical Analysis

The sample size was determined based on standard practices for genome-wide CRISPR screening and functional validation experiments, and no statistical power calculation was performed prior to the study. No outlier data were excluded from the analyses. Data are presented as mean ± SD or mean ± SEM, as specified in the corresponding figure legends. Statistical significance between two groups was determined using an unpaired two-tailed Student’s *t*-test. For pharmacological validation of reporter responses, differences among multiple groups were analyzed using one-way analysis of variance (ANOVA) followed by Dunnett’s multiple comparisons test, with the mock-treated group as the control. Associations between categorical variables were assessed using Fisher’s exact test or the hypergeometric test. Correlation analysis was performed using Pearson’s method. A *p*-value < 0.05 was considered statistically significant. All statistical analyses were performed using GraphPad Prism 10 software. Significance levels are denoted as * *p* < 0.05, ** *p* < 0.01, *** *p* < 0.001, and *ns* for not significant.

## 5. Conclusions

In summary, this study establishes a dual-fluorescence CRISPR screening framework for the systematic identification of candidate regulators of APP processing. By integrating genome-wide screening with biochemical validation and clinical transcriptomic analysis, we identified functional modules associated with metabolism, protein modification, and vesicular trafficking, and prioritized *LDHB*, *PIAS2*, *CCDC53*, and *TRIM61* as candidate modulators of APP proteolytic processing. These findings provide a useful resource for future mechanistic investigation and validation in more disease-relevant AD models.

## Data Availability

The datasets presented in this study can be found in SRA: PRJNA1439820. All other data supporting the findings of this study are available within the Article or Supplementary Materials.

## Supplementary Materials

The following supporting information can be downloaded at: https://www.mdpi.com/article/10.3390/ijms27093926/s1.

## Abbreviations

The following abbreviations are used in this manuscript:

Aβ	Amyloid-β
AD	Alzheimer’s disease
ADAM10	A Disintegrin and metalloproteinase domain-containing protein 10
AICD	APP intracellular domain
AMP-AD	Accelerating Medicines Partnership-Alzheimer’s Disease
APP	Amyloid precursor protein
BACE1	β-site APP cleaving enzyme 1
CRISPR	Clustered regularly interspaced short palindromic repeats
ER	Endoplasmic reticulum
FACS	Fluorescence-activated cell sorting
GO	Gene Ontology
iPSC	Induced pluripotent stem cell
MAGeCK	Model-based Analysis of Genome-wide CRISPR-Cas9 Knockout
MOI	Multiplicity of infection
NGS	Next-generation sequencing
OE	Overexpression
PCA	Principal component analysis
PPI	Protein–protein interaction
PS1	Presenilin 1
PS2	Presenilin 2
PTM	Post-translational modification
RRA	Robust Rank Aggregation
sAPPα	Soluble APPα
sgRNA	Single-guide RNA
UAS	Upstream Activating Sequence
UPR	Unfolded protein response
