# Supplementary material for: Genome-Wide CRISPR Screening Identifies Genetic Modulators of Amyloid Precursor Protein Processing
Source: Int J Mol Sci. 2026 Apr 28;27(9):3926. doi: 10.3390/ijms27093926 (PMC13163791; doi:10.3390/ijms27093926)
Supplement: Supplementary file 1 [file ijms-27-03926-s001.zip › Supplementary Materials-R1.pdf]

Supplementary Materials

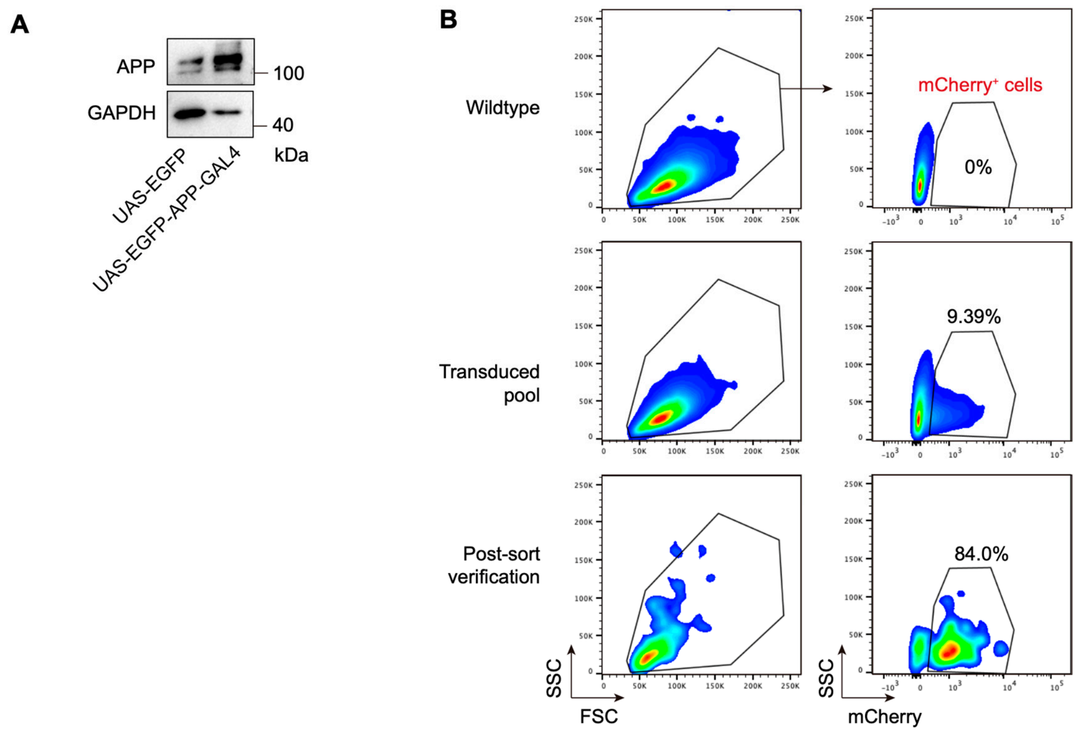

Supplementary Figure S1. Validation of the dual-fluorescence reporter system. **(A)** Representative immunoblot confirming the stable expression of the human APP-GAL4 fusion sensor protein in the engineered HEK293FT cell line, compared to the empty vector control. GAPDH serves as the loading control. **(B)** Flow cytometry-based gating strategy and validation for the generation of the monoclonal reporter cell line. Compared to the wild-type control (top), the initial transduced pool exhibited a 9.39% mCherry-positive population (middle). Following FACS-based single-cell sorting and clonal expansion, the post-sort verification confirmed robust and stable retention of the reporter construct, with the mCherry-positive population enriched to 84.0% (bottom).

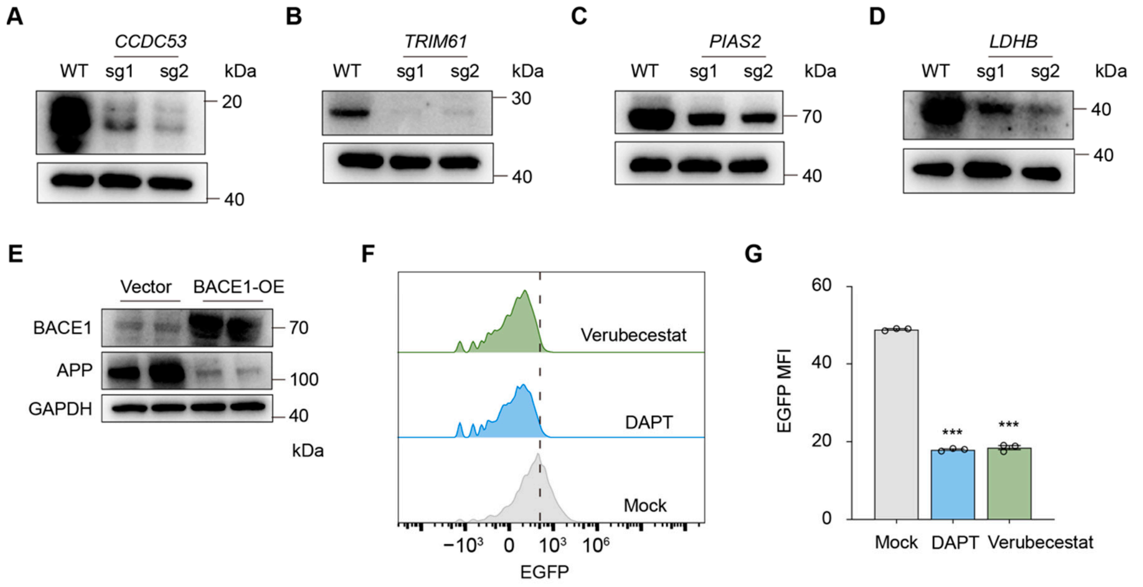

Supplementary Figure S2. Validation of candidate-gene perturbation, positive control, and pharmacological response of the APP-GAL4 reporter system. **(A-D)** Representative immunoblots demonstrating target protein depletion efficiencies. HEK293FT reporter cells were transduced with two independent sgRNAs (sg1, sg2) targeting *CCDC53* **(A)**, *TRIM61* **(B)**, *PIAS2* **(C)**, and *LDHB* **(D)** in HEK293FT reporter cells, compared with wild-type (WT) controls. **(E)** Immunoblot validation of the BACE1 overexpression (BACE1-OE) positive control model. Overexpression of BACE1 markedly increases BACE1 protein levels and concurrently reduces steady-state full-length APP substrate abundance. **(F)** Representative flow cytometry histograms showing reporter responses following treatment with the γ-secretase inhibitor DAPT or the BACE1 inhibitor verubecestat, compared with the mock control. **(G)** Quantification of EGFP mean fluorescence intensity (MFI) after pharmacological treatment. Both DAPT and verubecestat significantly reduced reporter signals relative to the mock control. Data are presented as mean ± SD (n = 3). Statistical significance was determined by one-way ANOVA followed by Dunnett's multiple comparisons test, with the mock group as the control (***$p < 0.001$).
